# Poly(Vinylpyrrolidone) Graft in Poly(Vinyl Chloride) Catheters Using Gamma Radiation for Ciprofloxacin Loading and Release

**DOI:** 10.3390/polym17050612

**Published:** 2025-02-25

**Authors:** Pedro J. Vargas-Machado, Felipe López-Saucedo, Emilio Bucio

**Affiliations:** Departamento de Química de Radiaciones y Radioquímica, Instituto de Ciencias Nucleares, Universidad Nacional Autónoma de México, Circuito Exterior, Ciudad Universitaria, Mexico City 04510, Mexico; pjvargasmachado@gmail.com

**Keywords:** antibacterial, grafting-from, gamma-rays, polymerization, PVC catheters, vinyl copolymers

## Abstract

This study addresses the modification of poly(vinyl chloride) catheters with *N*-vinylpyrrolidone and ciprofloxacin to achieve an antimicrobial surface. The copolymer was synthesized using the grafting-from method with gamma rays as a physical initiator and under different reaction conditions (absorbed dose, monomer concentration, and solvent). The modified catheters attained hydrophilic properties and were tested for ciprofloxacin loading and release efficiency. Antibiotic-loaded materials successfully inhibited the growth of *S. aureus* and *P. aeruginosa* strains. Therefore, surfaces with PVP chains exhibit suitable features for the loading and release of small molecules like ciprofloxacin (a fluoroquinolone). Results suggest that graft copolymers are suitable materials for the fabrication of biomedical devices with antibacterial features.

## 1. Introduction

Polymer materials are widely used in various biomedical fields, including implants, prostheses, pacemaker parts, suture threads, gauze, and catheters [1,2]. Catheters are used to drain fluids and administer electrolytes and drugs. These devices are typically made of poly(vinyl chloride) (PVC), polyurethane (PU), or silicone. PVC has excellent mechanical properties, making it ideal for catheter production. However, prolonged use may lead to bacterial biofilm formation [3], which can result in infections, affecting a significant percentage of patients and increasing mortality rates [4].

One approach to preventing the proliferation of pathogenic microorganisms through the catheter is the surface modification of these biomaterials with antimicrobial properties [5]. One method is “grafting-from” copolymerization, in which a polymer is modified with a monomer to form a second polymer attached to the surface (or in bulk), thereby introducing peripheral organic functional groups of interest [6,7]. The copolymerization process can be carried out using ionizing gamma rays [8,9,10,11]. These alternative energy sources may replace chemical initiators or catalysts in some reactions, thereby reducing the need for additional reagents and minimizing waste.

Polymer surfaces with appropriate functional groups can be used as loading and delivery systems. For example, hydrophilic copolymers containing OH, CO, or N groups [12] can function as drug delivery systems since most bioactive compounds (drugs) have polarizable functional groups and are usually non-covalently charged [13]. Therefore, quinolone antibiotics, such as ciprofloxacin [14], can be incorporated into antibacterial systems [15].

In this work, the grafting of *N*-vinylpyrrolidone (NVP) monomer onto PVC urinary catheters was achieved using gamma radiation as the initiator of the copolymerization reaction. The parameters influencing the grafting degree were studied to determine adequate reaction conditions. The modified material was evaluated using different characterization techniques to study its physicochemical and mechanical properties. In addition, the loading and release behavior of ciprofloxacin was analyzed under physiological conditions. Finally, antimicrobial assays were conducted to assess the inhibitory capacity of the catheter/antibiotic system against *Pseudomonas aeruginosa* and *Staphylococcus aureus* strains.

## 2. Materials and Methods

### 2.1. Reagents and Solvents

Poly(vinyl chloride) (PVC) catheters with a diameter of 3.30 mm and a thickness of 0.80 mm were obtained from Spectra Hardware Inc. (Westmoreland, PA, USA). The monomer *N*-vinylpyrrolidone (NVP) (≥99% GC) and ciprofloxacin (≥98%) were obtained from Aldrich Chemical (Saint Louis, MO, USA) and purified by distillation under reduced pressure. The solvents, anhydrous ethyl alcohol, and isopropyl alcohol were purchased from REPROQUIFIN Reactivos y Productos Químicos Finos (Ecatepec, State of Mexico, Mexico), and methanol was purchased from JT Baker (Phillipsburg, NJ, USA). All solvents were used undistilled.

For the antimicrobial tests, tubes with brain heart broth, tubes with Luria broth, and plates with Hinton Müeller Agar were used, all from the BD Bioxón™ kit (Franklin Lakes, NJ, USA). The bacterial strains used in the study were *Pseudomonas aeruginosa* ATCC™ 27853 and *Staphylococcus aureus* ATCC™ 25923, hereinafter *P. aeruginosa* and *S. aureus*, respectively.

### 2.2. Method for Obtaining PVC-g-PVP by Gamma Radiation

PVC catheters were cut into 3.0 cm-long fragments, washed with absolute ethanol, and dried in a vacuum oven at 313.15 K for 8 h. After determining each sample’s initial weight (Wi), the pieces were placed in glass tubes to prepare the ampoules used as containers during irradiation as mentioned below.

The synthesis of PVC-g-PVP was carried out using gamma radiation from a ^60^Co source using the direct method. A series of experiments were carried out varying different parameters (solvent, gamma irradiation dose, and monomer concentration) to find the most suitable conditions for grafting.

**Figure 1:**
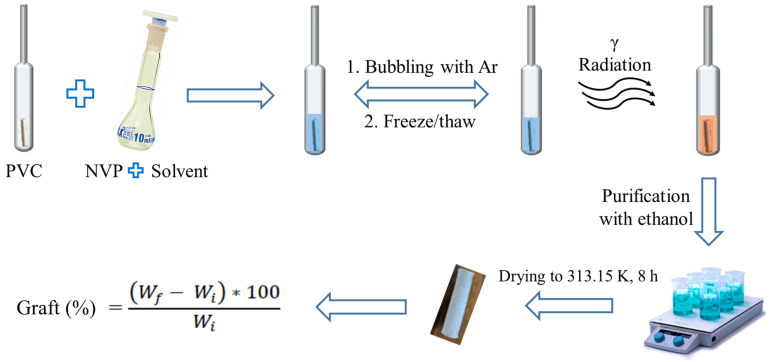
Experimental representation for the synthesis of PVC-g-PVP obtained via gamma radiation-induced grafting.

In the first step, the solvent and the NVP monomer were poured into the open ampoule containing the sample. Subsequently, oxygen was removed by air displacement through bubbling with argon for 15 min for the experiment with water solvent. In the experiments with the other solvents, oxygen was removed through several vacuum freeze/thaw cycles using liquid nitrogen in a closed system with a vacuum line. The ampoules were sealed and irradiated at a predetermined dose to start the graft polymerization process. The resulting copolymer was carefully extracted from the ampoule to avoid damaging it and washed with ethanol to remove any possible residues of homopolymer and non-grafted monomer. These samples were dried at 313.15 K in a vacuum oven for 8 h. Finally, the weight (*W_f_*) of each material obtained was determined, and the graft percentage was calculated by gravimetry using Equation (1). The experimental procedure described above can be seen in Figure 1.

(1) $$\mathrm{Graft}\;(\%)=\frac{W_{f}-W_{i}}{W_{i}}*100$$

where:

- $W_{f}$ is the final weight of the grafted copolymer.
- $W_{i}$ is the initial weight of the PVC.

#### 2.2.1. Reaction Conditions: Solvent

The solvent study was the first parameter evaluated to determine graft behavior concerning the other reaction variables, specifically the effect of dose and monomer concentration. Various solvents (water, methanol, ethanol, and isopropanol) were used to investigate the graft performance of NVP on PVC catheters. These tests were carried out using a 50% *v*/*v* NVP solution and a 50 kGy irradiation dose.

#### 2.2.2. Reaction Conditions: Absorbed Dose

Doses between 5 and 70 kGy (5, 10, 20, 30, 40, 50, 60, and 70 kGy) were applied to irradiate the PVC samples in the NVP solution. For this experiment, isopropanol was chosen as a solvent at a concentration of 50% *v*/*v*.

#### 2.2.3. Reaction Conditions: NVP Concentration

The samples were irradiated with a dose of 50 kGy, with concentrations of NVP between 20 and 70% *v*/*v* (20, 30, 40, 50, 60, 70% *v*/*v*) and using isopropanol as solvent.

### 2.3. Swelling Tests

The weight of dry PVC and PVC-g-PVP samples was recorded. The samples were swelled in distilled water at 298.15 K. Every 15 min, the sample was extracted, excess water was removed with a wipe, and the samples were weighed. Once completed 60 min after the first measurement, the weights were measured every 30 min until the weight remained constant. Each experiment was carried out in triplicate.

The swelling was also performed in phosphate buffer solution (PBS) at pH 7.4 at 310.15 K, following the methodology described above. The swelling percentage was calculated using Equation (2).

(2) $$\mathrm{Swelling}\;(\%)=\frac{W_{s}-W_{d}}{W_{d}}*100$$

where:

- $W_{s}$ is the final weight of the swollen sample.
- $W_{d}$ is the weight of the dry sample.

### 2.4. Contact Angle

Glass plates were used to flatten the catheters and measure their wettability. The treatment applied to these samples to determine the contact angle is described in the Supplementary Materials in Figure S1. Once the surfaces were flat, a drop of distilled water was deposited, and the contact angle between the surface and the liquid was measured using the camera built into the contact angle measuring device. Measurements were taken in quadruplicate in various areas of the catheters, both on the outside and inside.

### 2.5. Preparation of Samples for Drug Loading and Release

#### 2.5.1. Ciprofloxacin Loading

The loading of the ciprofloxacin was carried out for samples of approximately 100 mg on grafted and pristine PVC catheters. The samples were placed in amber vials with 5 mL of an aqueous solution with ciprofloxacin 9 µg/mL for 48 h at 298.15 K. The loading of the drug in the catheter was evaluated by reading absorbance with a UV-VIS spectrophotometer at 266 nm at 1, 2, 4, 6, 24, 30, and 48 h. These experiments were performed with quartz cuvettes, 1 cm in length.

Calibration curve: A standard solution of ciprofloxacin was prepared between 9.0 µg/mL and 0.9 µg/mL. Measurements were performed at room temperature and in triplicate to obtain Equation (3), where *A* represents absorbance and C is the concentration of ciprofloxacin (µg/mL).

(3) $$A=0.102C+0.069\;R^{2}=0.999$$

#### 2.5.2. Ciprofloxacin Release

For drug release, the loaded samples were placed in amber vials with 5 mL of PBS solution at pH 7.4 and a temperature of 310.15 K to simulate physiological conditions. Samples were kept under mechanical stirring at 100 rpm. Absorbance at 266 nm was recorded at 0.5, 1, 2, 4, 6, 24, 30, 48, and 54 h. Measurements were performed in triplicate.

The calibration curve was performed in PBS at pH 7.4 as a medium at 310.15 K, using ciprofloxacin concentrations between 0.1 and 5 µg/mL. The measurements were performed in triplicate, and Equation (4) was obtained, where *A* is absorbance and *C* is the concentration of ciprofloxacin (µg/mL).

(4) $$A=0.198C+0.081\;R^{2}=0.999$$

### 2.6. Antimicrobial Testing

#### 2.6.1. Hinton Müeller Agar Dishes Preparation

Petri dishes were prepared with Hinton agar by adjusting the pH of the medium with 1.0 M HCl(aq) or 1.0 M NaOH(aq), respectively. The medium at different pH was sterilized in an autoclave at 394.15 K for 15 min. Once the medium reached 318.15 K, the agar was placed in Petri dishes for solidification. The Petri dishes were placed in a 308.15 K incubator for 24 h before the sterility test.

#### 2.6.2. Preparation of Strains

Strains *S. aureus* ATCC 25923 and *P. aeruginosa* ATCC 27853 that were freeze-dried were activated with brain heart broth or Luria broth for 24 h. Subsequently, 100 µL of each microorganism strain was taken and inoculated into the media for 7 h. Afterwards, an aliquot was taken with a Pasteur pipette and added drop by drop to its respective fresh culture medium until the microbial density was adjusted to the McFarland (MF) standard of 0.5.

#### 2.6.3. Concentration of Bacteria Used in the Test

The quantity of bacteria in each suspension (0.5 MF) was calculated using the surface extension technique. From the bacterial suspension adjusted to 0.5 MF, 6 consecutive dilutions were made in the tubes with isotonic saline solution. Of the last 3 concentrations (1 × 10^−4^, 1 × 10^−5^, and 1 × 10^−6^), 100 µL were taken and placed in Petri dishes with the Hinton Müeller agar. The inoculum was spread using sterile glass beads and allowed to absorb. The dishes were incubated at 308.15 K for 24 h. Once the incubation period was over, the number of colonies was counted, and the concentration found was 182 × 10^6^ bacteria/mL for *S. aureus* and 127 × 10^6^ bacteria/mL for *P. aeruginosa*, respectively.

#### 2.6.4. Antimicrobial Inhibition Assay

A sample of the 0.5 MF standard solution was taken with a swab and spread over the surface of the culture medium in the Petri dish. This procedure was carried out in 3 Petri dishes for each strain. The samples were placed according to the distribution indicated in the diagram in Figure S2. The dishes were incubated at 308.15 K for 24 h. After the incubation time, the inhibition halos of each sample were measured with a vernier caliper.

### 2.7. Instrumental

Source of irradiation

Cobalt-60 irradiator (^60^Co Gammabeam 651-PT) of the Institute of Nuclear Sciences of the National Autonomous University of Mexico. Irradiation intensity of 8.43 kGy/h.

FTIR-ATR

Perkin-Elmer Spectrum 100 from Perkin Elmer Cetus Instruments (Norwalk, CT, USA), with an ATR module. A total of 16 scans were made in the range of 4000 to 650 cm^−1^.

TGA

Approximately 15 mg of the dried samples were analyzed at 313.15 K. A platinum balance was used with a TGA Q500 from TA Instruments (New Castle, DE, USA). The study was performed under a nitrogen atmosphere with a heating ramp of 283.15 K/min from 293.15 to 1073.15 K.

SEM

PVC-g-PVP samples with different graft percentages and the pristine PVC were cut into pieces of approximately 1 cm^2^, which were dried in a vacuum oven at 313.15 K for 8 h. A JEOL model 5900-LV scanning electron microscope (SEM) (Musashino, Akishima, Tokyo, Japan) was used. Images were taken at different magnifications (100×, 200×, and 1000×) to visualize the surface and cross-section.

Contact angle

The equipment used was a DSA Krüss GmBH goniometer (Hamburg, Germany).

Mechanical tests

The catheter samples were cut into 3 cm and flattened for tensile testing on a Shimadzu Autograph universal testing machine, model AGS-X (Tokyo, Japan). The distance between samples was 10 mm, and the analysis was performed at 10 mm/min. Each sample was performed in quadruplicate.

UV-VIS Spectrophotometer

SPECORD 200 Plus UV-Vis spectrophotometer from Analytik Jena AG, (Jena, Germany), and 1 cm long quartz cuvettes were used.

Software: Origin Pro 9.0 and Excel 16.0 with DDSolver.

## 3. Results

### 3.1. Synthesis of PVC-g-PVP Using Gamma Rays

**Figure 2:**
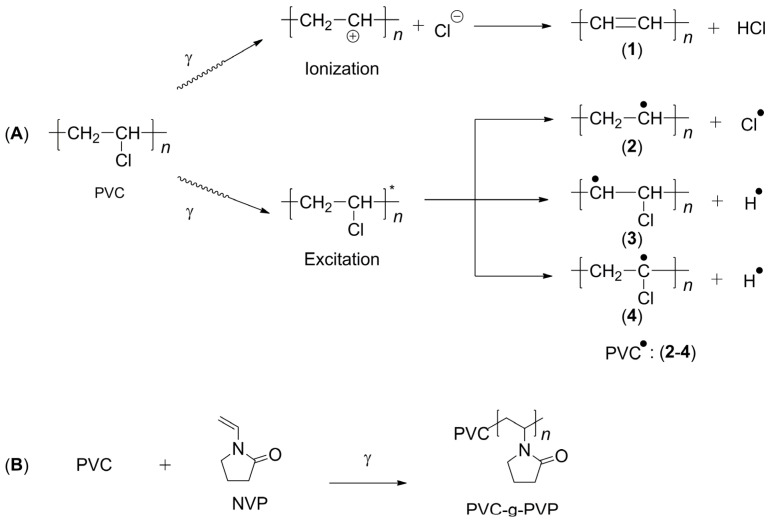
(**A**) Ionization (+) and excitation (*) processes induced by gamma radiation in PVC. (**B**) Grafting reaction for the synthesis of the copolymer (PVC-g-PVP).

The grafted polymer called PVC-g-PVP was obtained using gamma radiation by the direct method, also called simultaneous irradiation [16]. The species generated during irradiation are shown in Figure 2A [17]. When the PVC molecule is irradiated, the C-Cl bond is more likely to be broken, which generates a free radical on the C atom [18,19], which acts as a starting point for polymerization. Figure 2B shows a proposal for the reaction mechanism resulting from the grafting process between PVC and NVP.

In this method, the reaction conditions affect the grafting percentage, mainly the solvent used, irradiation dose, and monomer concentration.

The solvent effect was investigated using water, methanol (MeOH), ethanol (EtOH), and isopropanol (*i*PrOH) in a 50% *v*/*v* concentration with the NVP monomer at a dose of 50 kGy; the grafting results are shown in Table 1.

The results show that the highest graft yields were obtained using methanol (149%) and ethanol (134%). However, the catheters with the highest graft percentages were damaged, indicating that these reaction conditions were unsuitable. In contrast, using water as a solvent did not facilitate graft copolymer formation. Isopropanol proved to be the most suitable solvent, yielding an intermediate graft of 78.5% without damaging the catheters. Figure S3 illustrates the effects of different solvents on graft yield and catheter integrity.

**Figure 3:**
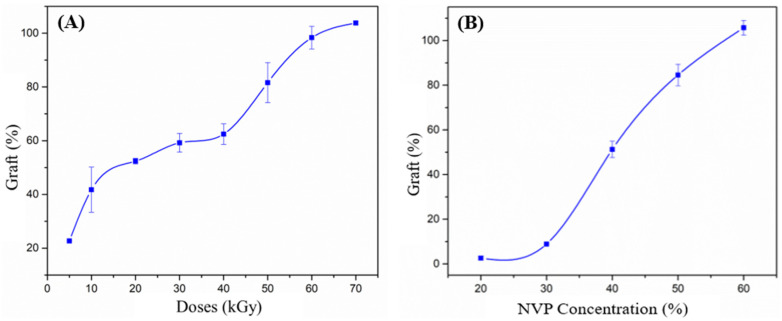
NVP grafting series on PVC in isopropanol as the solvent. (**A**) NVP grafting at different absorbed doses (50% *v*/*v* NVP). (**B**) Grafting of NVP at varying monomer concentrations (50 kGy).

Experiments varying the dose from 5 to 70 kGy indicate that the percentage of grafting increases as the irradiation dose increases. Figure 3A shows an upward trend; however, the polymer deteriorates for doses above 50 kGy, and the material becomes rigid. Also, at high doses, a large amount of homopolymer is formed, which makes catheter extraction difficult since the polymer contained in the ampoule solidifies. Therefore, doses between 10 and 40 kGy seem more suitable under these reaction conditions.

The concentration of the NVP monomer in an isopropanol solution was studied within the range of 20–70% *v*/*v*. The percentage of grafting increased as the NVP concentration increased, as observed in Figure 3B. However, at a concentration of 70% *v*/*v*, the catheter dissolved, preventing the determination of grafting. At an NVP concentration of 60% *v*/*v*, the grafting exceeded 100%. Conversely, at low NVP concentrations (20 and 30% *v*/*v*), the grafting was minimal. Therefore, intermediate concentrations (40 and 50% *v*/*v*) are more suitable for obtaining acceptable graft copolymers.

### 3.2. IR-ATR Spectroscopy

**Figure 4:**
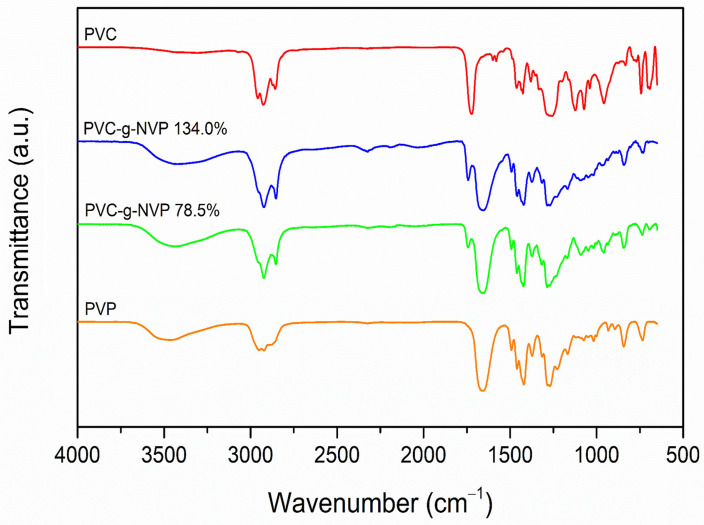
FTIR-ATR spectra of PVC, PVP, and PVC-g-PVP (78.5 and 134%).

The grafting of NVP monomer onto PVC was confirmed by infrared spectroscopy. The graph in Figure 4 shows the spectra of PVC, PVP, and two PVC-g-PVP samples with different grafting percentages.

The pristine PVC exhibited relevant vibrational bands: one at 2926 cm^−1^ due to the stretching of the CH bond from the methylene groups (CH_2_), another at 1723 cm^−1^ due to the plasticizer, two bands at 1256 and 1463 cm^−1^ due to the bending of the CH bond, and one at 693 cm^−1^ due to the stretching of the C-Cl bond [20].

The PVP homopolymer showed a stretching band at 2950 cm^−1^ that belongs to the CH bond of the CH_2_ group and the band of the carbonyl group (C=O) at 1656 cm^−1^. It also appears the CH asymmetric deformation bending at 1420 cm^−1^ that corresponds to the CH_2_ group. Finally, the stretching CN bond at 1283 cm^−1^ is observed [21].

The bands in the IR spectrum of the modified materials resemble those observed in the PVC and PVP spectra, respectively. In the spectra of the modified materials, the band corresponding to the carbonyl group appears at 1659 cm^−1^ for both samples PVC-g-PVP (78.5% graft) and PVC-g-PVP (134.0% graft). Whereas the band associated with the stretching of the CN bond is located at 1286 cm^−1^ for PVC-g-PVP (78.5% graft) and at 1285 cm^−1^ for PVC-g-PVP (134.0% graft). However, in the spectra of the modified materials, the bands corresponding to PVC appear more intense than the C=O and the CN bands from the PVP-grafted chains.

### 3.3. TGA of Polymers

**Figure 5:**
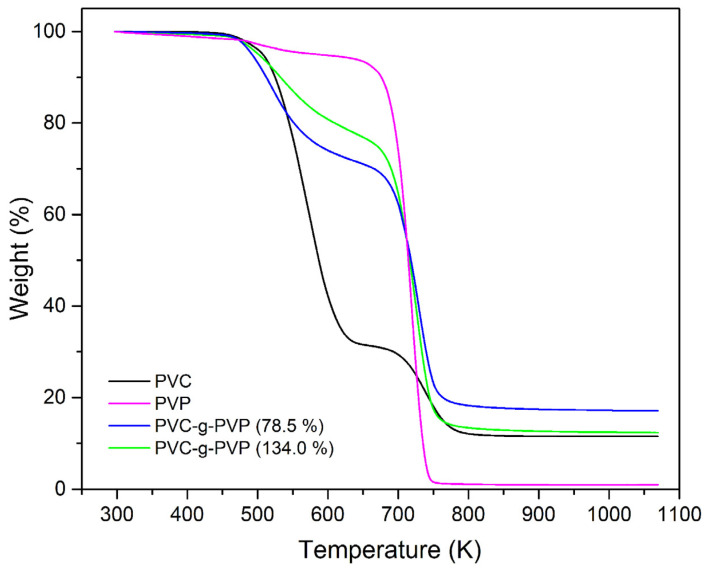
TGA of PVC, PVP, and PVC-g-PVP (78.5 and 134.0%) in an N_2_ atmosphere heated up to 1073.15 K.

The thermal behavior of PVC-g-PVP was determined using TGA by analyzing two different grafted samples and comparing them with PVC and PVP polymers, as shown in Figure 5. Pristine PVC exhibits a 10% weight loss at 524.15 K and three decomposition temperatures at 527.88, 571.94, and 740.45 K. The first decomposition temperature corresponds to the plasticizer, the second to the dehydrochlorination of PVC, and the third to the complete decomposition of the polymer chains [6]. Meanwhile, PVP exhibited a 10% weight loss at 676.10 K and a decomposition temperature of 718.66 K.

The copolymer materials PVC-g-PVP (78.5%) and PVC-g-PVP (134.0%) presented a 10% weight loss at 512.35 and 532.62 K, respectively; these temperatures are similar to the observed in the PVC thermogram. The first decomposition temperature of PVC-g-PVP (78.5%) was 520.20 K, and PVC-g-PVP (134.0%) was 527.71 K, respectively; and the second decomposition temperatures of copolymers were 455 and 726.15 K, respectively, coinciding with the homopolymer PVP decomposition temperature (Table 2). Finally, all materials containing PVC showed a residue above 11% weight at 1073.15 K due to silicon oxides, SiO and SiO_2_ [22].

### 3.4. Swelling and Contact Angle Tests

**Figure 6:**
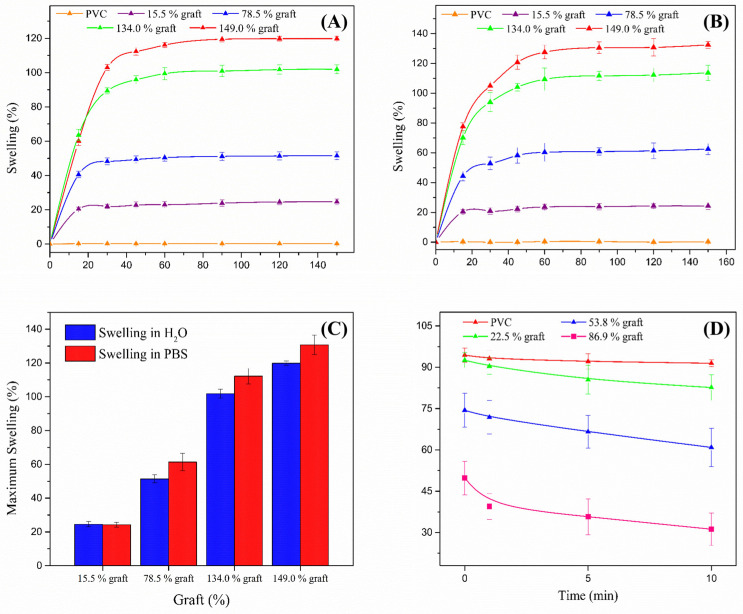
(**A**) Swelling limit in distilled water at 298.15 K for samples with different PVP graft percentages. (**B**) Swelling limit in PBS at pH 7.4 and 310.15 K for samples with different PVP graft percentages. (**C**) Swelling limit of samples with different PVP graft percentages in water at 298.15 K and PBS at 310.15 K. (**D**) Contact angle behavior at 0, 5, and 10 min.

Swelling tests conducted in distilled water at 298.15 K demonstrated that the modified materials are hydrophilic [23]. In general, swelling increases with a higher graft percentage, whereas PVC does not swell. The swelling of the grafted catheters reaches equilibrium at 120 min, after which the material’s weight remains constant. Figure 6A shows a maximum swelling of 120% for the material with the highest graft percentage.

The same pattern was observed in the swelling experiments in PBS solution at pH 7.4 and 310.15 K, where a gradual increase in swelling respecting graft percentage was observed up to 130.7% weight, which also was reached at 120 min and corresponded to the highest graft percentage measured (Figure 6B). In general, the swelling values obtained for each sample follow the tendency of those observed in distilled water, although the swelling percentages in PBS are higher (Figure 6C).

The contact angle of a water droplet was also used to determine surface hydrophilicity [24,25]. For hydrophilic materials, the measured contact angles are less than 90°. Conversely, in hydrophobic materials, the water droplet is repelled by the surface, resulting in contact angles greater than 90°. Figure 6D presents the contact angle measurements of PVC, PVC-g-PVP (22.5%), PVC-g-PVP (53.8%), and PVC-g-PVP (86.9%) at 0, 5, and 10 min. Surface wettability increased with the PVP graft, consistent with the swelling analysis (Table S1).

### 3.5. Scanning Electron Microscopy (SEM)

**Figure 7:**
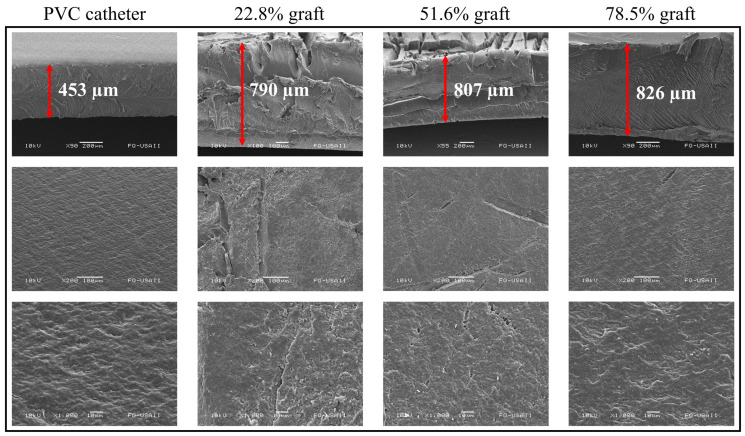
SEM images of PVC and PVC-g-PVP catheters with grafting degrees of 22.8, 51.6, and 78.5%.

SEM analyses were performed for PVC, PVC-g-PVP (22.8%), PVC-g-PVP (51.6%), and PVC-g-PVP (78.5%). Figure 7 shows 3 rows, with the samples analyzed from different perspectives and magnifications.

The first row corresponds to different samples seen from the cross-section. In these images, an increase in thickness of 75, 78, and up to 83% can be observed in the three grafted materials compared to the pristine PVC. Due to the shearing effect caused by the cross-section, some images are not observed beyond the thickness acquired due to the graft.

Rows two and three of Figure 7 correspond to the catheter surface at 200× and 1000×, respectively. Even in the pristine PVC, a surface with some roughness is observed. In comparison, the grafted materials have a fractured surface in the middle section. Perhaps due to radiation wear or the grafted polymer itself. Nonetheless, the polymer with the highest grafting degree has a surface comparable to pristine PVC.

### 3.6. Mechanical Properties of Catheters

**Figure 8:**
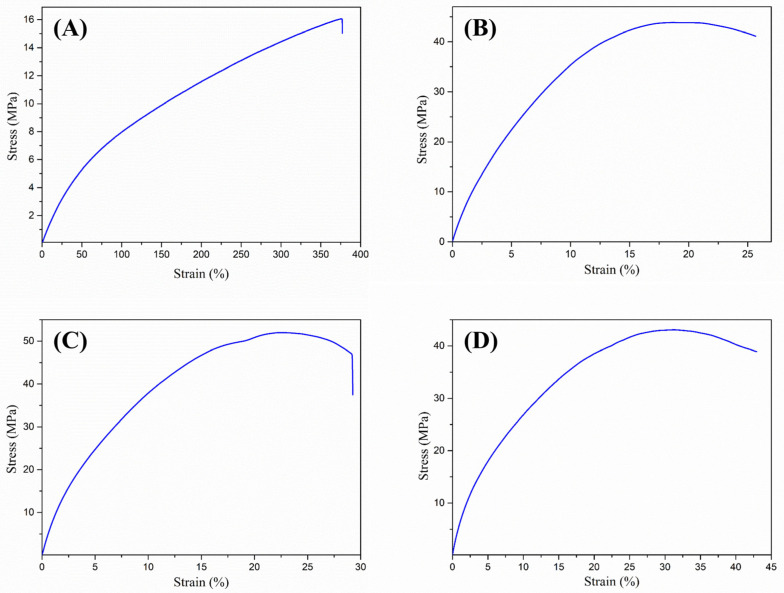
Stress (MPa) vs. Strain (%) diagram of pristine PVC (**A**) and PVC-g-PVP with grafting degree of (**B**) 22.5%, (**C**) 53.8%, and (**D**) 86.9%.

The mechanical properties of a material are crucial for its effective performance in its intended application. Figure 8A presents the mechanical behavior of pristine PVC, showing a maximum deformation of approximately 397.65 ± 26.00% and a maximum stress of 16.47 ± 1.01 MPa at the breaking point. Based on Young’s modulus, these values indicate that pristine PVC is well-suited for use as a catheter due to its high flexibility and strength.

Figure 8B–D corresponds to samples of PVC-g-PVP with graft percentages (B) 22.5, (C) 53.8, and (D) 86.9%. It is observed that the radiation and the grafting degree of NVP cause changes in the mechanical properties of the PVC catheters (Table S2). These changes are an increase in stress and a decrease in the percentage of deformation, which corresponds to polymers such as grafted catheters that have a stiffer appearance than the pristine material.

### 3.7. Loading and Release of Ciprofloxacin

After characterizing the PVC-g-PVP copolymers, the loading and release of ciprofloxacin were studied. Drug release was conducted in PBS at pH 7.4 and 310.15 K to simulate physiological conditions.

**Figure 9:**
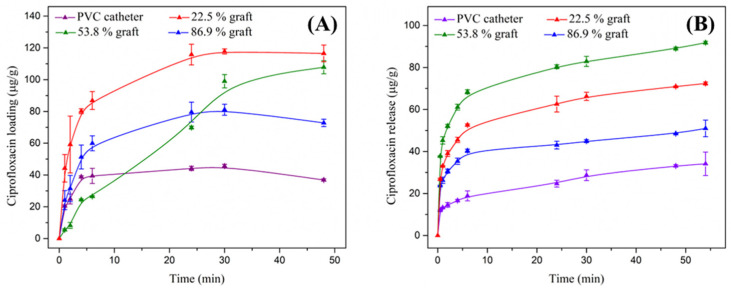
(**A**) Ciprofloxacin load curves of the catheter materials PVC and PVC-g-PVP (22.5, 53.8, and 86.9%). (**B**) Ciprofloxacin release curves of the catheter materials PVC and PVC-g-PVP (22.5, 53.8, and 86.9%).

Loading curves of PVC control, PVC-g-PVP (22.5%), PVC-g-PVP (53.8%), and PVC-g-PVP (86.9%) showed that the material with the highest loading capacity was the catheter with the lowest graft (22.5%), with approximately 116 µg/g (Figure 9A). In the case of pristine PVC, a loading capacity of less than 40 µg/g was presented. Regarding the other two grafts (53.8 and 86.9%), the ciprofloxacin loading values were 108 and 73 µg/g, respectively (Table S3). The PVP graft (22.5%) tripled the loading capacity of PVC, which is favorable for drug loading. However, among the grafted materials, the load-bearing capacity decreases with increasing grafting degree, possibly because the bulk grafting exposes PVC chains mixed with PVP chains on the surface.

Ciprofloxacin release tests were measured up to 54 h. Figure 9B shows that PVC-g-PVP (53.8%) released the highest amount of ciprofloxacin, 92 µg/g. While PVC-g-PVP (22.5%) released 72 µg/g and PVC only released 34 µg/g. In addition, data analysis was performed to determine the release kinetics of the system. It was found that the ciprofloxacin release curve best fits the Peppas-Sahlin (R^2^ = 0.997) and Korsmeyer-Peppas (R^2^ = 0.997) models (Tables S4–S7), which correspond to polymeric release systems [26].

### 3.8. Antibacterial Performance

The Kirby–Bauer method was performed for grafted samples with 22.8, 44.3, and 78.5% and loaded with ciprofloxacin [27], using as a control the respective unloaded copolymers and the pristine PVC. Microbiological tests were performed in agar cultures with *S. aureus* and *P. aeruginosa*.

**Figure 10:**
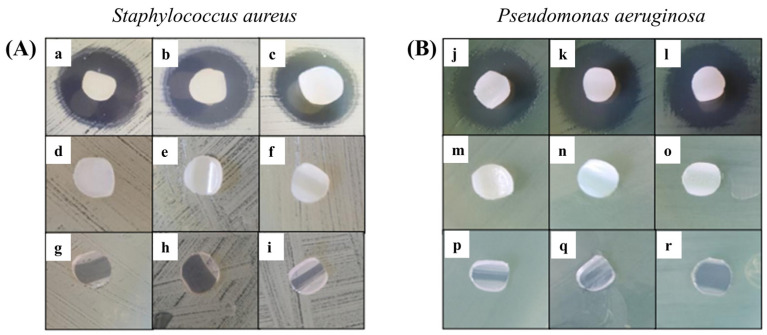
Inhibition zones in *S. aureus* (**A**) and *P. aeruginosa* (**B**) cultures for PVC-g-PVP samples loaded with ciprofloxacin, with grafting percentages of 22.8% (**a**,**j**), 44.3% (**b**,**k**), and 78.5% (**c**,**l**). Controls PVC-g-PVP with grafting percentages of 22.8% (**d**,**m**), 44.3% (**e**,**n**), and 78.5% (**f**,**o**) and PVC (**g**–**i**,**p**–**r**).

It was observed that all PVC-g-PVP and ciprofloxacin-loaded materials showed inhibition (Figure 10). The first row of both figures (Figure 10A,B) belongs to the modified catheters loaded with ciprofloxacin in increasing order of grafting. The second row contains the controls for the drug-free grafted materials, and the last row also corresponds to the controls for pristine PVC catheters. Furthermore, it was determined that inhibition was greater in *P. aeruginosa* strains, with halos measuring an average of 20 mm. Meanwhile, the inhibition halos of *S. aureus* strains measured an average of 17 mm.

## 4. Discussion

*About grafting*: The results of NVP grafting onto PVC catheters to obtain PVC-g-PVP suggested gamma radiation-induced grafting as a suitable method to obtain the copolymer [28]. However, not all reaction conditions produce grafts. Based on the grafting experiments, it is concluded that the best conditions for obtaining graft copolymers are irradiation doses between 5 and 40 kGy, NVP monomer concentrations of 40 and 50% v/v, and using isopropanol as a solvent.

*Ciprofloxacin loading/release*: The increased hydrophilicity of the copolymers arises from polar intermolecular forces between carboxylic groups, amines, and water, which are the same interactions occurring between water (or PBS) and ciprofloxacin during the loading and release processes [29]. The release percentages indicate that the drug concentration is sufficient to achieve bacterial inhibition. It was also found that unmodified PVC loaded less ciprofloxacin but released 92%, likely due to weaker drug-polymer interactions. PVC-g-PVP (22.5%) exhibited the lowest release percentage, likely due to stronger drug-polymer interactions resulting from surface grafting [30]. Meanwhile, PVC-g-PVP (53.8%) released the highest amount of ciprofloxacin, possibly because bulk grafting increases the presence of PVC groups on the surface. Despite the high grafting degree, this effect weakens drug-polymer interactions [31].

*Antibacterial properties*: The halos in the antibiograms of both strains are within the intermediate range of inhibition, that is, between susceptible and resistant. The results are encouraging since the drug concentration is lower than that used by the standard reference, which is 5 µg [32]. Therefore, increasing the concentration of ciprofloxacin may improve antimicrobial activity [27].

## 5. Conclusions

The grafting of the NVP monomer into PVC catheters using gamma radiation made it possible to obtain a biomaterial that favors the loading of drugs and their subsequent release at concentrations that enable bacterial growth inhibition. The optimal conditions to obtain the copolymer PVC-g-PVP were isopropanol as a solvent, with doses between 5 kGy and 40 kGy, and NVP concentrations of 40 and 50% *v*/*v*. According to the contact angle and swelling studies in water and PBS, the PVC-g-PVP grafts are hydrophilic. This property was used to make loading and release systems with the antibiotic ciprofloxacin. Tests with bacterial cultures *P. aeruginosa* and *S. aureus* showed that the modified ciprofloxacin-loaded systems inhibited both types of cultures. Although further studies are required, it can be concluded that PVP-grafted surfaces appear viable for manufacturing antimicrobial sanitary disposables.

## Figures and Tables

**Table 1:** Effect of solvent on the degree of grafting.

Solvents	MeOH	EtOH	*i*PrOH	H_2_O
Graft (%)	149.0	134.0	78.5	0.7

**Table 2:** Data obtained from thermogravimetric analysis.

Material	10% Weight Loss (K)	Decomposition Temperature (K)	Residue at 1073.15 K (%)
PVC	524.84	527.88, 571.94, 740.45	11.52
PVP	676.10	718.66	0.97
PVC-g-PVP (78.5%)	512.35	520.20, 728.90	17.15
PVC-g-PVP (134%)	532.62	527.71, 726.59	12.37

## Data Availability

The original contributions presented in the study are included in the article, further inquiries can be directed to the corresponding authors.

## Supplementary Materials

The following supporting information can be downloaded at: https://www.mdpi.com/article/10.3390/polym17050612/s1, Figure S1: Treatment of samples for contact angle; Figure S2: Arrangement of the inhibition experiment in disc cultures; Figure S3: Grafting using different solvents; Table S1: Contact angles; Table S2: Mechanical tests; Table S3: Release of ciprofloxacin; Tables S4–S7: Release models.
