# Supplementary material for: Poly(Vinylpyrrolidone) Graft in Poly(Vinyl Chloride) Catheters Using Gamma Radiation for Ciprofloxacin Loading and Release
Source: Polymers (Basel). 2025 Feb 25;17(5):612. doi: 10.3390/polym17050612 (PMC11902714; doi:10.3390/polym17050612)
Supplement: Supplementary file 1 [file polymers-17-00612-s001.zip › polymers-3483211-supplementary.pdf]

Article

Poly(Vinylpyrrolidone) Graft in Poly(Vinyl Chloride) Catheters Using Gamma Radiation for Ciprofloxacin Loading and Release

Pedro J. Vargas-Machado, Felipe López-Saucedo * and Emilio Bucio *

Departamento de Química de Radiaciones y Radioquímica, Instituto de Ciencias Nucleares, Universidad Nacional Autónoma de México, Circuito Exterior, Ciudad Universitaria, Mexico City 04510, Mexico; pjvargasmachado@gmail.com
* Correspondence: felipelopezsaucedo@gmail.com (F.L.-S.); ebucio@nucleares.unam.mx (E.B.)

Academic Editor: Asterios (Stergios) Pispas

Received: 1 February 2025
Revised: 21 February 2025
Accepted: 23 February 2025
Published: 25 February 2025

Citation: Vargas-Machado, P.J.; López-Saucedo, F.; Bucio, E. Poly(Vinylpyrrolidone) Graft in Poly(Vinyl Chloride) Catheters Using Gamma Radiation for Ciprofloxacin Loading and Release. *Polymers* **2025**, *17*, x. https://doi.org/10.3390/xxxxx

Copyright: © 2025 by the authors. Submitted for possible open access publication under the terms and conditions of the Creative Commons Attribution (CC BY) license (https://creativecommons.org/licenses/by/4.0/).

1. Supplementary

S.1. Experimental arrangements

S.1.1. Treatment of samples for contact angle

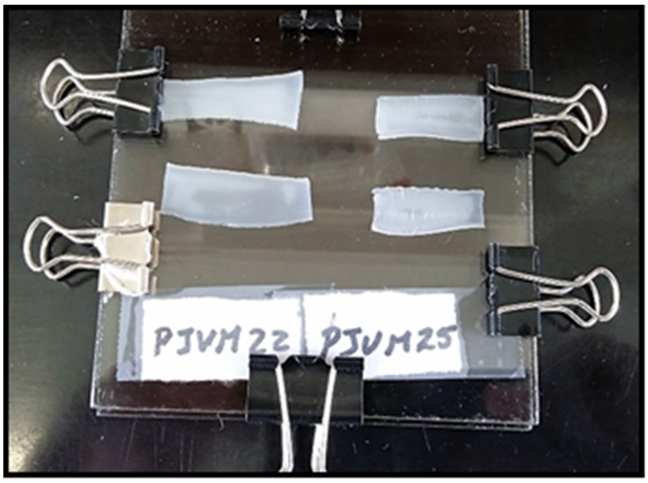

Figure S1. PVC and grafted samples between a pair of glass plates after drying and flattening process in a vacuum oven for 8 h at 40 °C. Samples were prepared for contact angle measurement.

S.1.2. Arrangement of the inhibition experiment in disc cultures

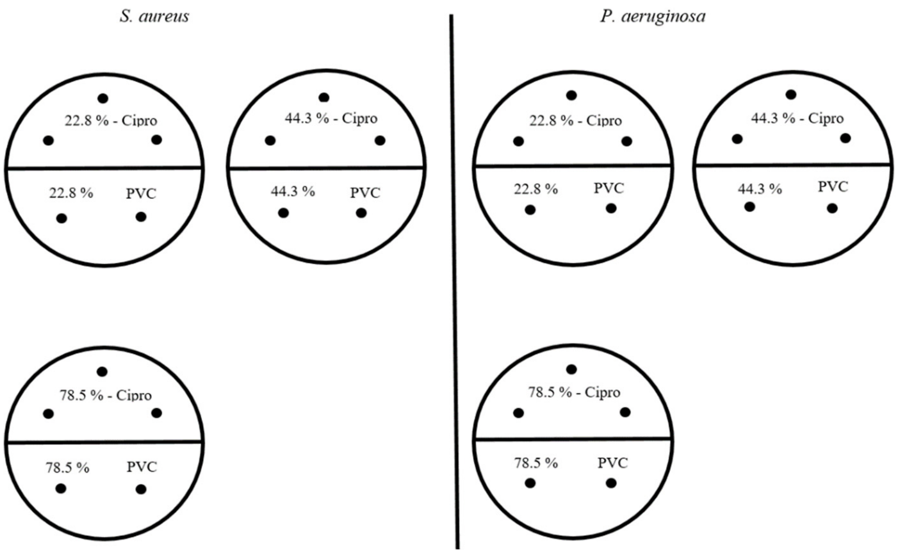

Figure S2. Distribution diagram for the analyzed samples in antimicrobial tests. The samples labeled as -Cipro were loaded with ciprofloxacin, whereas X% corresponds to the PVP grafting degree (X% = 22.8, 44.3, or 78.5). The controls do not have the word -Cipro indicated.

S.2. Results

S.2.1. Grafting using different solvents

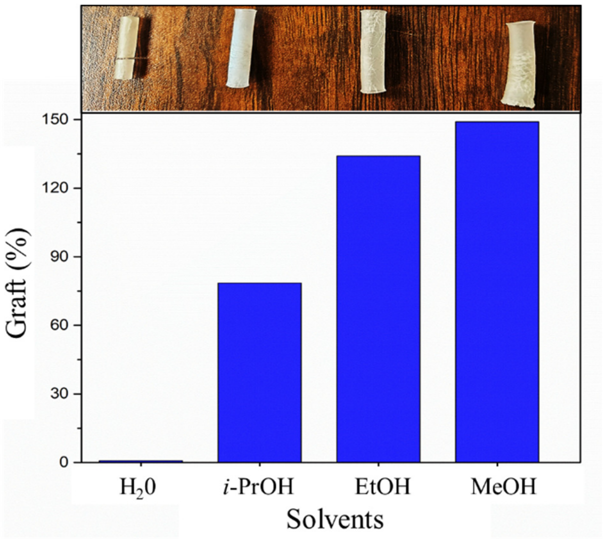

Figure S3. Grafted samples of PVP onto PVC using different solvents (**down**); and the appearance of those samples (**up**).

S.2.2. Contact angles

Table S1. shows the contact angles and images of samples under study, measured at 10 minutes after the water droplet fell on the surface. Contact angles at a time higher than 10 minutes could not be measured.

Samples	Angle (°)	Image
PVC	91.5 ± 1.3	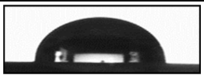
PVC-g-PVP (22.5%)	82.7 ± 4.6	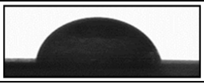
PVC-g-PVP (53.8%)	60.9 ± 6.9	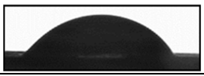
PVC-g-PVP (86.9%)	31.2 ± 5.9	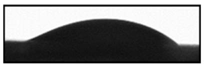

S.2.3. Mechanical tests

Mechanical test values are presented in **Table 2S**, including those of Young's Modulus.

Table S2. Tensile test results for the samples studied.

Samples	Young's modulus (MPa)	Maximum stress (MPa)	Maximum Strain (%)
PVC	14.34 ± 0.26	16.47 ± 1.01	397.65 ± 26.00
PVC-g-PVP (22.5%)	702.64 ± 23.19	44.35 ± 1.89	18.36 ± 2.68
PVC-g-PVP (53.8%)	845.25 ± 78.93	52.02 ± 1.19	23.00 ± 0.93
PVC-g-PVP (86.9%)	585.96 ± 53.77	43.16 ± 2.16	31.29 ± 2.08

S.2.4. Release of ciprofloxacin

Table S3. Data obtained for the load, release and percentage of release at the final time of each study in the analyzed materials.

Sample	Loading (μg/g)	Release (μg/g)	Release (%)
PVC	36.88 ± 0.73	34.14 ± 5.59	92.57
PVC-g-PVP (22.5%)	116.50 ± 5.26	72.36 ± 0.70	62.11
PVC-g-PVP (53.8%)	107.82 ± 4.14	91.78 ± 0.69	85.12
PVC-g-PVP (86.9%)	72.79 ± 2.30	50.98 ± 3.95	70.04

S.2.5. Release models

The release systems were adjusted for Higuchi, First order, First order with Tag and Fmax, Korsmeyer-Peppas, and Peppas-Sahlin. For the fitting of these models DDSolver

software in Excel were used. The parameters (R^2), Akaike Information Criterion (AIC) and Model Selection Criterion (MSC) were determined, results are shown in **Tables 4S-7S**.

Table S4. Release models and fitting parameters of the selected release models for PVC-g-PVP (22.5%).

**Release models**	**R^2**	**AIC**	**MSC**
Higuchi	0.911	80.927	-0.254
First order	0.885	86.634	-0.825
First order with Tag and Fmax	0.961	64,899	1.348
Korsmeyer-Peppas	0.997	39.168	3.922
Peppas-Sahlin	0.997	29.083	4.506

Table S5. Release models and fitting parameters of the selected release models for PVC-g-PVP (53.8%).

**Release models**	**R^2**	**AIC**	**MSC**
Higuchi	0.890	87,528	-0.586
First order	0.953	78.715	0.2956
First order with Tag and Fmax	0954	70.682	1.099
Korsmeyer-Peppas	0.997	42.581	3.909
Peppas-Sahlin	0.997	32.655	4.415

Table S6. Fitting parameters of the selected release models for PVC-g-PVP (86.9%).

**Release models**	**R^2**	**AIC**	**MSC**

Higuchi	0.856	77,686	-0.977
First order	0.778	84,540	-1.662
First order with Tag and Fmax	0.948	59,478	0.844
Korsmeyer-Peppas	0.982	33.662	2.892
Peppas-Sahlin	0.986	33,551	2.904

According to the selection criteria evaluated, the mathematical model that best describes the release process for all materials is the one developed by Peppas-Sahlin (**Equation S1**). This model has the R^2 close to 1, the lowest AIC, and the highest MSC.

$$\frac{M_t}{M_\infty} = k_1 t^m + k_2 t^{2m} \qquad \text{(S1)}$$

Where:

$\frac{M_t}{M_\infty}$: is the fraction of drug released; M_t is the amount of drug released at time t, and M_∞ corresponds to the amount of drug in the equilibrium.

k_1: is the constant of the Fickian diffusion mechanism.

k_2: is the constant of relaxation of polymer chains.

m: is a Fickian diffusion exponent for a material of any geometry that exhibits controlled release.

Table S7. shows the values corresponding to the constants of the model developed by Peppas-Sahlin for the samples analyzed. The results indicate that the diffusion mechanism follows Fickian behavior.

Table S7. Constants of the Peppas- Sahlin model for the samples studied.

**Samples**	k_1	k_2	m
PVC-g-PVP (22.5%)	37.886 ± 0.045	-4.813 ± 0.014	0.285 ± 0.002
PVC-g-PVP (53.8%)	52.782 ± 0.090	-7.413 ± 0.029	0.262 ± 0.002
PVC-g-PVP (86.9%)	32.354 ± 0.085	-5.009 ± 0.088	0.0225 ± 0.003
